# Cardiorenal Effects of Kappa Opioid Peptides During Ontogeny

**DOI:** 10.3390/ph4010154

**Published:** 2011-01-11

**Authors:** Francine G. Smith, Wei Qi

**Affiliations:** Department of Physiology & Pharmacology/Paediatrics, Faculty of Medicine, University of Calgary, 3330 Hospital Drive NW, Calgary, T2N 4N1, Canada

**Keywords:** kappa opioid peptides, KORs, newborn, physiology

## Abstract

This review focuses on the physiological roles for kappa opioid receptors (KORs) in adult animals and humans, as well as in the developing newborn animal. Our recent findings have provided new information that under physiological conditions in conscious newborn animals, activation of KORs with the selective agonist, U-50488H, results in an aquaresis, as previously observed in adult animals and humans. In addition, we have shown in conscious lambs that KORs modulate systemic and renal haemodynamics as well as the arterial baroreflex control of heart rate, providing a previously unidentified role for KORs.

## 1. Introduction

Endogenous opioid peptides, also known as endorphins, are part of the endocrine system, and are involved in numerous physiological processes including feeding, cardiovascular and endocrine function. As well, endorphins are involved in pain, substance abuse, traumatic brain injury, and haemorrhagic shock [1]. The known effects of endorphins are elicited through dynorphins, β-endorphins, endomorphins, enkephalins, and nociceptins/orphanin FQ which activate specific receptor subtypes designated as kappa (κ) opioid receptor (KOR), mu (μ) opioid receptor (MOR), and delta (δ) opioid receptor (DOR), as well as the N/OFQ peptide receptor (NOP). This review focuses on the putative roles for the first class of endorphins – the dynorphins – and their cardiovascular and renal effects through activation of KORs, especially with regard to the physiology of development.

## 2. Dynorphins

**Figure 1:**
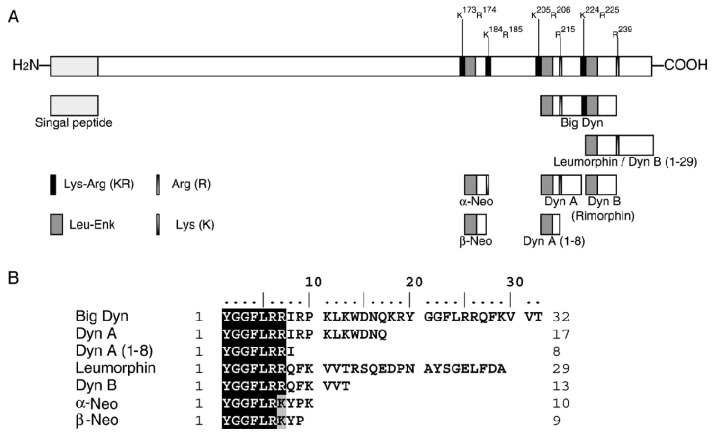
Generation and amino acid sequences of naturally occurring dynorphins.

In the late 70s and early 80s, several kappa preferring endogenous peptides were identified, their common precursor being prodynorphin (PDYN) which was subsequently cloned in 1982 by Akil *et al.* [1]. Seven peptides are generated from PDYN by sequential enzymatic cleavage mainly through proprotein convertase 2 (Figure 1) [2]. PDYN-derived peptides collectively known as dynorphins are putative endogenous ligands for KORs and comprise dynorphin A (Dyn A), dynorphin A(1-8), dynorphin B (Dyn B), α-neoendorphin (α-Neo), β-neoendorphin (β-Neo), leumorphin, and big dynorphin (Big Dyn, which contains both Dyn A and Dyn B) (Table 1) (Figure 1) [3]. The potential of dynorphin to bind to and activate KORs is similar to that of synthetic κ-agonists such as ethylketocyclazocine (EKC), bremazocine, and U-50488H. Dynorphins can also bind to MOR and DOR, although their selectivity for KOR is considerably greater which can be attributed to differences in the structure of the KOR second extracellular loop which contains eight amino acids and displays an amphiphilic helical region, which is not present in MOR or DOR [4]. Dyn A has a 46-fold higher affinity for KOR than Dyn B whilst the affinity of Big Dyn for KOR is similar to Dyn A because the binding potency correlates with the presence of the Dyn A(9-17) segment (see Table 1), present in both Big Dyn and Dyn A but lacking in Dyn A(1-18) and Dyn B which demonstrate lower potency [3].

## 3. Cardiovascular Effects of KOR

Previous studies in adult mammals have explored the role of KOR centrally in modulating cardiovascular homeostasis: Dynorphin when microinjected into the preoptic *medialis nucleus* of anaesthetised rats results in a decrease in both arterial pressure and heart rate [5] and microinjection into the *nucleus tractus solitarius* (NTS) of the selective KOR agonist, U-50488H, elicits a dose-dependent increase in arterial pressure and a decrease in heart rate similar to that observed following injection of dynorphin [6]. Furthermore, U-50488H microinjected into the parvocellular paraventricular nucleus (PVN) results in an immediate pressor response along with a bradycardia [7].

In contrast, the actions of KOR agonists administered into the peripheral circulation are not completely understood [8]. For example, systemic administration of the KOR agonists bremazocine, tifluaodom, and U-50488H to urethane-anaesthetised Sprague Dawley rats is associated with a decrease in arterial pressure [9]. In chloralose-anaesthetised cats, spiradoline mesylate administered systemically decreases arterial pressure and in baroreceptor-denervated cats, there is a dose-related hypotensive effect [10]. In healthy human volunteers, I.V. infusion of the KOR agonist, niravoline, induces a transient increase in arterial pressure but no concomitant change in heart rate [11]. In conscious adult ewes, Szeto *et al.* showed that I.V. administration of U-50488H, also increases arterial pressure as well as heart rate [12]. To date, there have been no studies investigating the role of kappa opioid peptides in regulating the arterial baroreflex. Taken together then, the aforementioned studies investigating systemic cardiovascular effects of KOR agonists in adult animals and humans have provided variable and often conflicting results likely resulting from differences in experimental design including state of consciousness, dose, choice of drug, and mode of administration.

## 4. Renal Effects of KORs

Initially, opiate-like activity was identified in kidney tissues [13,14]; however, it is now apparent that there are considerable species differences in the intra-renal localization of specific ORs [15,16]. In the opossum kidney (OK) cell line, a model of proximal tubular cells, a high affinity KOR subtype (κ_1_) has been identified [17]. This is known as the KOR1 and is believed to be the receptor through which the endogenous ligand, dynorphin A, as well as the structurally similar nociceptin, elicit their renal effects [18] as detailed in the proceeding paragraphs:

For almost three decades, it has been known that KORs are involved in fluid homeostasis. Both central and peripheral administration of selective KOR agonists as well as the endogenous ligand, dynorphin, leads to an increase in urinary flow rate also known as a diuresis, in rats [19-23], mice [24], dogs [25], and humans [26,27]: Slizgi and Ludens first reported a diuretic response to subcutaneous (S.Q.) administration of the partial KOR agonist and benzomorphan analogue, EKC to conscious rats [28]. Leander described a diuresis following S.Q. administration of bremazocine, EKC, and ketazocine, which was inhibited by the selective KOR antagonists, WIN-44,441 and MR-2266BS [20]. In hydrated conscious rats, Hiudobro-Toro and Parada measured a diuretic response to intra-peritoneal (I.P.) administration of KOR agonists; this diuresis was abolished by the selective KOR antagonists (WIN-44,441 and MR2266BS) but not the non-selective antagonist, naloxone [19]. Dykstra *et al.* [29] also reported a dose-dependent diuretic response to S.Q. administration of U-50488H in conscious rhesus monkeys. The diuresis was abolished by the selective KOR antagonist, MR-2266. In human subjects, Kramer *et al.* [27] showed that administration of the KOR agonist, asimadoline, was also associated with a diuretic response.

KOR agonists and the naturally occurring ligands, dynorphins, also increase free water clearance and therefore are considered aquaretic compounds [30-32]. This aquaresis is thought to result from a combination of its direct inhibitory effects on the tubular action of arginine vasopressin (AVP) in promoting water reabsorption, and suppression of AVP release from the neurohypophysis [21,23,31,33-36]. With respect to the latter, KOR activation within the central nervous system (CNS) is associated with stimulation of the PVN concomitantly with a triggering of brainstem noradrenergic regions that innervate this nucleus [37].

There is also evidence that KOR and AVP work synergistically within specific brain neurons: Dynorphin is co-localized with AVP and KOR in neurosecretory vesicles that are exocytosed from dendrites and terminals of cells within the supraoptic nucleus (SON). In hypothalamo-neurohypophysial explants of Long Evans rats, Rossi *et al.* [38] showed that AVP gene expression, as well as osmotically-induced AVP secretion were inhibited by the KOR agonist, RU 51599, at the level of the hypothalamus. In neurosecretosomes removed from Wistar rat pituitaries, Zhao *et al.* [39] showed that U-50488H and dynorphin A_1–13_ inhibited AVP secretion evoked by direct depolarization of the nerve terminals, thus providing evidence that KORs are located on the nerve terminals of magnocellular neurons with direct modulation by kappa opioid peptides.

Brown *et al.* [40] showed that in SON cells of female Sprague Dawley rats, administration of the selective KOR antagonist, Nor-BNI, increases the activity of phasically active, vasopressinergic neurons. This demonstrates an auto-inhibitory effect of dynorphin at the level of vasopressin cells of the SON, or presynaptically on their afferent inputs. The same group using intracellular recordings of SON cells in superfused hypothalamic explants of male Long Evans rats and showed that Nor-BNI alters burst patterns and removes silent periods between bursts resulting in a continuous firing pattern [41]. Therefore, it appears that dendritic dynorphin release contributes to the termination of spontaneous phasic bursts *in vivo* in magnocellular neurosecretory cells of the SON.

A role for κ-opioid peptides in influencing tubular electrolye reabsorption in the adult has also been implicated, although the literature in this regard is inconsistent: Huidobro-Toro and Parada demonstrated a dose-dependent anti-natriuresis following I.P. administration of U-50488H to hydrated conscious rats [19], but no consistent anti-kaliuretic effect. In the isolated perfused rat kidney, however, administration of the KOR agonist, ketocyclazocine, has no effects on sodium or potassium excretion rates [42]. Kapusta and Obih [43] showed that U-50488H administered I.C.V. elicited a decrease in sodium excretion which occurs concomitantly with an increase in renal sympathetic nerve activity; this anti-natriuresis is abolished in the absence of renal sympathetic nerves. Ashton *et al.* [32] also measured an anti-natriuresis and anti-kaliuresis following S.Q. injection of U-50488H to inactin-anesthetized rats. In contrast, an increase in sodium excretion occurred following I.V. administration of U-50488H to pentobarbital-anesthetized dogs [25], whereas administration of the stable dynorphin analog, E2078, to conscious rats elicited no change in sodium excretion yet potassium excretion decreased [44].

Taken together, it is generally well recognized that kappa opioid peptides are aquaretic. To date, however, their role in tubular electrolyte reabsorption has not been fully characterized. With the recent advancement in new technologies including novel ligands and cellular, molecular, and genetic techniques, it will be important for new studies to further evaluate the renal effects of kappa opioid peptides and therefore help to increase our understanding of their role in regulating fluid and electrolyte homeostasis.

## 5. Physiological Effects of KORs during Development

**Figure 2:**
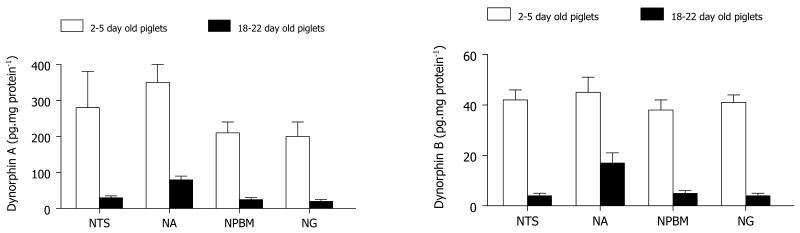
Dynorphin levels in brainstem regions of developing piglets.

Elevated levels of enzymes such as enkephalin convertase (which forms enkephalin) around the time of birth in rats predict a heightened opioid activation in the newborn [45]. Zhang and Moss [46] demonstrated an age-related increase in the content of β-endorphin, methionine-enkephalin, as well as dynorphin A and B within the NTS, ambigualis, gigantoreticularis and parabrachialis medialis nuclei in piglets (Figure 2). These are brain regions that normally influence cardiorespiratory control. In the ovine brain, maximum opioid receptor binding is present in the pons and medulla by term of gestation, remaining high in newborn lambs and decreasing with postnatal maturation [47]. Specifically, KOR binding sites as well as their density increase gradually with postnatal development in the rat [48-51], guinea pig [48,49], mouse [52], and human [53] in various organs including the brain, spinal cord and heart, implicating alterations in their physiological roles during ontogeny. Therefore, it seems likely that κ-opioid peptides may modulate cardiovascular and renal function during the perinatal period.

## 6. Cardiovascular Effects of KOR during Development

**Figure 3:**
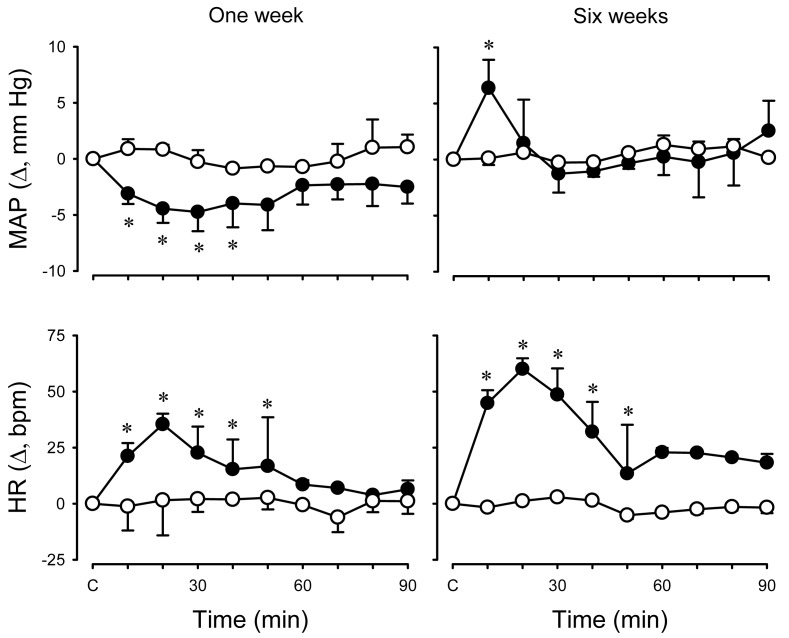
Effects of U-50488H on mean arterial pressure (MAP) and heart rate (HR) in conscious lambs.

**Figure 4:**
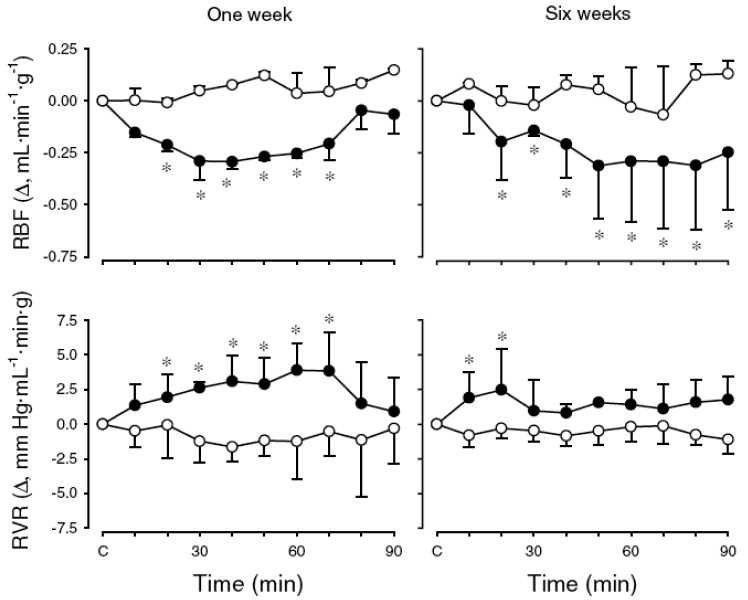
Effects of U-50488H on renal blood flow (RBF) and renal vascular resistance (RVR) in conscious lambs.

To test the hypothesis that activation of KORs modulates systemic and renal haemodynamics in an age-dependent manner, cardiovascular effects of the KOR agonist, U-50488H, were investigated in conscious lambs at two stages of post natal maturation and under physiological conditions [54]. First, experiments were undertaken to determine the maximum effective dose (ED100) of U-50488H to be applied in subsequent experiments. Cardiovascular measurements were made for 30 min before and 90 min after I.V. injection of U-50488H over the range of doses 0-5.0 mg/kg. In both age groups of lambs, the U-50488H dose *versus* peak heart rate response curve was constructed and the ED100 determined to be 0.5 mg/kg at one week and 1.0 mg/kg at six weeks [54]. In subsequent experiments, the aforementioned ED_100_ dose of U-50488H was administered to conscious lambs and shown to elicit a small but sustained decrease in mean arterial pressure one week post natally whereas a small but transient increase occurred at six weeks (Figure 3). In both age groups of lambs, there was a sustained increase in heart rate after U-50488H (Figure 3). There was also a significant and sustained decrease in renal blood flow in both age groups following I.V. administration of U-50488H, resulting from an increase in renal vascular resistance (Figure 4). Although the mechanism underlying this renal haemodynamic response to U-50488H is not known, it may reflect an increase in renal sympathetic nerve activity [55,56], although effects of local vasoactive factors cannot be ruled out.

**Figure 5:**
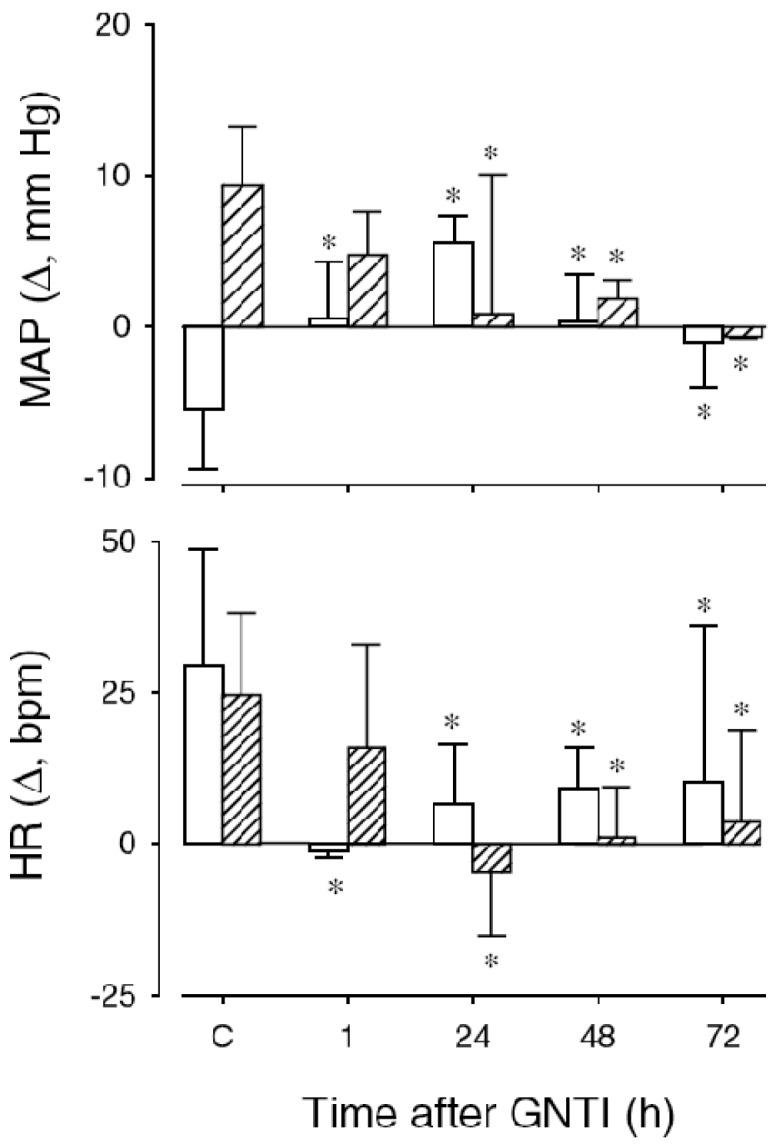
Effects of GNTI on mean arterial pressure (MAP) and heart rate (HR) responses to U50488H in conscious lambs.

To investigate whether the aforementioned responses to U-50488H resulted from direct activation of KORs and to rule out any non-receptor or secondary effects of U-50488H, additional experiments were carried out in the presence of the selective KOR antagonist 5′guanidinyl-17-(cyclopropylmethyl)-6,7-dehydro-4,5α-epoxy-3,14-dihydroxy-6,7-2′,3′-indolomorphinan dihydrochloride (GNTI). A potent KOR antagonist, GNTI displays 208- and 799-fold selectivity over MOR and DOR, respectively, and greater antagonist potency than the prototypical KOR antagonist, norbinaltorphimine, (nor-BNI) [57,58]. As illustrated in Figure 5, the maximum inhibitory effects of GNTI on the cardiovascular responses to U-50488H are present within 24 h and remain for up to 72 h.

**Figure 6:**
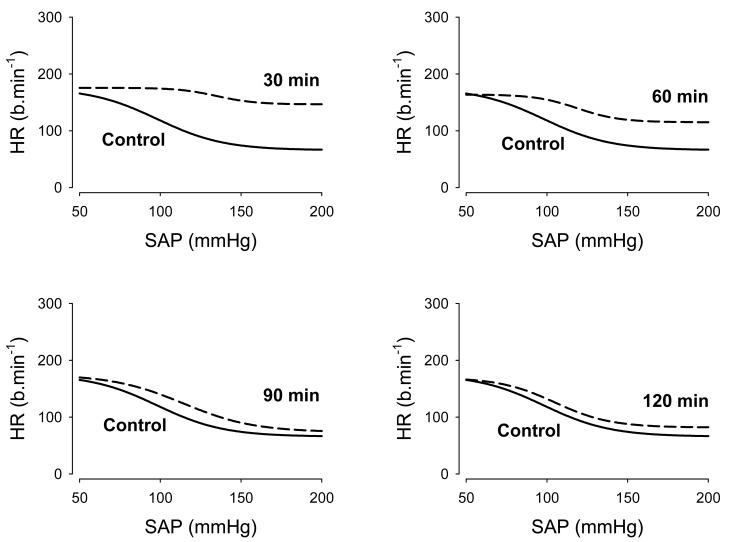
Activation of KOR modulates the arterial baroreflex control of heart rate (HR) in conscious lambs.

Because the KOR agonist, U50488H, increased heart rate (as described above), with minimal changes in pressure, it appeared that KORs might also modulate the arterial baroreceptor reflex. Therefore, in an ensuing study, we measured the parameters governing the arterial baroreflex control of heart rate in the presence and absence of U-50488H in conscious young lambs [59]. In this study, the dose of 5.0 mg/kg of U-50488H was selected as it produced a sustained increase in both arterial pressure and heart rate [59]. Administration of U-50488H considerably decreased the heart rate range (*P1*) through a dramatic increase in the minimum heart rate (*P4*) at 30 min. There was also an increase in the systolic arterial pressure at the midpoint of the heart rate range (*P3*) as well as the slope coefficient (*P2*) an increase in the maximum gain (*Gmax*) (Figure 6). Taken together, these findings provide the first direct evidence that κ-opioids may modulate the baroreflex, revealing a previously unidentified role for this opioid peptide.

Although all OR subtypes have been localized to midbrain and brainstem regions involved in cardiovascular integration, KOR binding sites predominate in the preoptic area, and in several hypothalamic regions of the rat brain including the supraoptic and paraventricular nucleus, notably the magnocellular and parvocellular regions [60]. There is also considerable KOR binding in the NTS, at least in the rat brain [61,62]. Laorden *et al.* [37] demonstrated increased c-*fos* expression in the rat paraventricular nucleus as well as noradrenergic A_1_ and A_2_ cell groups following I.P. administration of U-50488H. In addition, catecholaminergic-positive neurons in the NTS and ventrolateral medulla which innervate the paraventricular nucleus showed a significant increase in *fos* expression following U-50488H [37]. Therefore, the observed effects of U-50488H in modulating the arterial baroreflex control of heart rate could reflect activation of KOR in these regions of the CNS.

## 7. Renal effects of KOR during development

**Figure 7:**
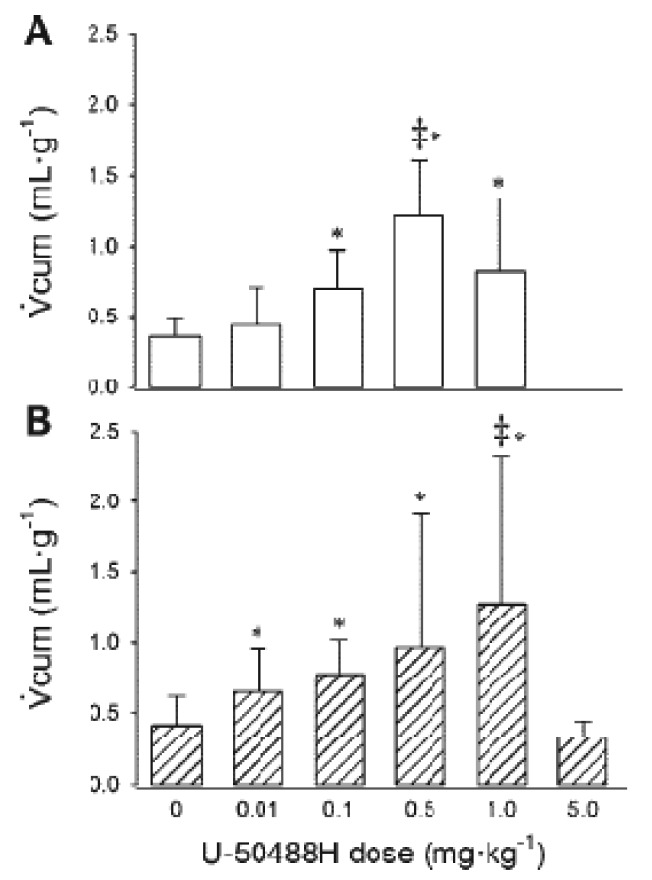
Dose dependent effects of U-50488H on cumulative urinary flow rate (Vcum).

The endorphin precursors proenkephalin A and B are detectable in the kidney of newborn rat pups but not adult rats [63,64] and newborn piglets but not adult pigs [65,66]. Little is known, however, regarding the renal responses to activation of KORs during the perinatal period, or whether they are in fact aquaretic during this time. Jackson and Kitchen [67] reported an increase in urinary output in response to I.P. administration of U-50488H to 10 day old rat pups, although no other measurements were made and no additional age groups were studied. Recently, Qi *et al.* measured renal responses to activation of KORs in conscious lambs [68]. Initially, the relationship between U-50488H dose and cumulative urinary flow rate (Vcum) was determined to define the maximal effective dose (ED_100_) for both age groups as 0.5 mg/kg (one week) and 1.0 mg/kg (six weeks) [68] (Figure 7).

**Figure 8:**
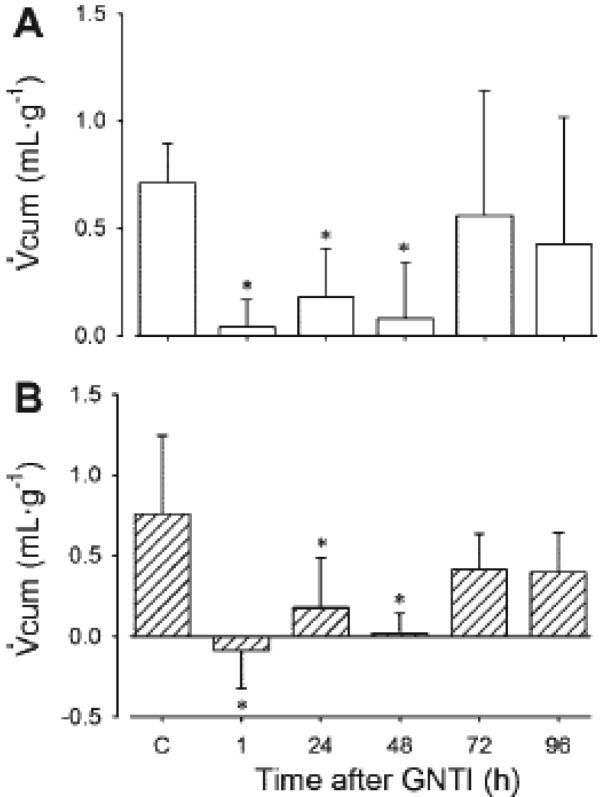
Effects of GNTI on Vcum responses to U-50488H in conscious lambs.

In subsequent experiments, ED100 U-50488H was administered and urinary flow rate and electrolyte excretions were measured. As for adult animals and humans, administration of U-50488H, to lambs aged ∼one-and ∼six weeks elicits a diuresis which is abolished by the selective KOR antagonist, GNTI. Since this diuretic response to U-50488H was abolished 1 h after pre-treatment with GNTI, with effects persisting for 48 h (Figure 8), we can conclude that it results from the direct action of KORs. The diuresis which followed administration of U-50488H to conscious lambs was unaccompanied by any alterations in electrolyte excretions. There was, however, an accompanying increase in free water clearance which provides evidence that the effects of KOR in the newborn period are in fact aquaretic.

In hypothalamic neurons in culture derived from 70-day gestation ovine fetuses, AVP is secreted basally and in response to K^+^-induced depolarizations [69]. Dynorphin also inhibits basal as well as K^+^-stimulated AVP release, demonstrating that the negative feedback control of AVP by activation of KORs is intact before birth [69] in the developing ovine. These findings provide evidence that the diuretic effect of KOR activation early in life may result from alterations in AVP release which could reflect age-dependent changes in the central distribution of KOR as described in section 5 above.

## 8. Pharmacological Inhibition of KORs

Previously, the prototypical KOR antagonist used in physiological studies has been nor-BNI. This compound, however, produces non-selective antagonism for both MOR and DOR 1-2 h after its administration [70-71]. Nor-BNI also has a relatively low potency *in vivo* after systemic administration, a slow onset and long duration of action, with effects persisting for many weeks. GNTI is a recently synthesized KOR antagonist which is considered more potent than nor-BNI [57,72]. In a schedule-controlled behavioral study in the rhesus monkey [73], GNTI exhibited antagonism of U-50488H-induced behavioural effects in a dose- and time-dependent manner. Negus *et al.* demonstrated that GNTI had a faster onset and shorter duration than nor-BNI, with a peak effect at 24 h after I.V. administration, with effects persisting for ∼four days [73]. Our experiments in conscious lambs provide the first physiological investigations into the role of GNTI as a tool to evaluate the contributions from KORs to cardiovascular and fluid and electrolyte homeostasis in the conscious animal. Because this compound is more potent and selective for KORs as compared to nor-BNI, it may be considered a more suitable pharmacological KOR antagonist for future experimental studies.

## 9. Conclusions

Our experiments under physiological conditions in conscious newborn animals have provided the first information that activation of KORs results in an aquaresis, as previously observed in adult animals and humans. In addition, KORs modulate systemic and renal haemodynamics as well as the arterial baroreflex control of heart rate, providing a previously unidentified role for KORs. It is apparent that kappa opioids are emerging as an important peptide under physiological conditions throughout life with important functions early in post natal life. Additional studies are clearly warranted to more fully elucidate the role that kappa opioid peptides may play in regulating electrolyte balance. New investigations are also needed into a role for KORs in regulating the arterial baroreflex. Future studies should also evaluate a role for KORs in such pathophysiological states as haemorrhagic shock, pain, and brain injury in the newborn which may have important clinical ramifications.

## Figures and Tables

A. Endogenous dynorphins are generated from the precursor through differential cleavages by proprotein convertases at paired and single basic amino acids followed by carboxypeptidases; B. Amino acid sequences of prodynorphin gene products. Reproduced from Chen *et al.* [4] with permission.

Dynorphin A (left) and dynorphin B (right) measured in brainstem regions of piglets. NTS, nucleus tractus solitarius; NA, nucleus ambiguus; NPB, nucleus parabrachialis; NG, nucleus gigantoreticularis. Values are mean ± S.E.M. Adapted from Zhang and Moss [46].

Mean arterial pressure (MAP, upper panel) and heart rate (HR, lower panel) measured before and after I.V. administration of vehicle (open symbols) or U-50488H (solid symbols) to conscious lambs aged ∼one week (left; N = 7) and ∼six-weeks (right; N = 10). * p < 0.05 compared to control (C). Reproduced from Qi and Smith [54] with permission.

RBF (upper panel) and RVR (lower panel) measured before and after I.V. administration of vehicle (open symbols) or U-50488H (solid symbols) to conscious lambs aged ∼one week (left hand panel; N = 7) and ∼six-weeks (right hand panel; N = 10). * p < 0.05 compared to control (C). Reproduced from Qi & Smith (2006) [54] with permission.

Change in MAP (top) and HR (bottom) after I.V. administration of U-50488H at 24 h (control, C) and at 1, 24, 48, and 72 h after I.V. injection of GNTI to lambs aged ∼one week (open bars, n = 4) and ∼six weeks (hatched bars, n = 4). p < 0.05 compared to C. Reproduced from Qi and Smith [54] with permission.

A logistic function was applied to pooled raw data for control measurements (solid line) and for 30 min, 60 min, 90 min, and 120 min, after administration of U-50488H (dashed line). Reproduced from Qi and Smith [59] with permission.

Urine flow rate was measured over 90 min (Vcum) after administration of U-50488H to conscious lambs aged ∼one week (A) and ∼six weeks (B). * p < 0.05 compared with 0 dose. ‡ indicates maximum effective dose (or ED100). Reproduced from Qi *et al.* [68] with permission.

Urine flow rate was measured over 90 min (Vcum) after administration of U-50488H before (Control, C) and at 1, 24, 48, 72, and 96 h after I.V. administration of GNTI to lambs aged ∼one week (A) and ∼six weeks (B). * p < 0.05 compared to C. Reproduced from Qi *et al.* [68] with permission.

**Table 1:** Amino acid sequences of dynorphins.

**Peptide**	**Amino Acids (n)**	**Sequence**
Big Dyn*	1-32	YGGFLRRI**RPKLKWDNQ**KRYGGFLRRDFKVVT
Dyn A(1-8)	1-8	YGGFLRRI
Dyn A	1-17	YGGFLRRI**RPKLKWDNQ**
Big Dyn(6-26)	6-26	RRI**RPKLKWDNQ**KRYGGFLRR
Dyn B	20-32	YGGFLRRDFKVVT

* human prodynorphin 207-238; Bolded, shadowed region indicates the amino acid sequence Dyn(9-17) which correlates to potency of binding of peptide to KOR. Adapted from Merg *et al.* [3].
